# Sulforaphane and ophthalmic diseases

**DOI:** 10.1002/fsn3.4230

**Published:** 2024-05-22

**Authors:** Yichi Zhang, Xiaojing Zhao, Yang Liu, Xiuxia Yang

**Affiliations:** ^1^ Department of Ophthalmology The Fifth Affiliated Hospital of Sun Yat‐sen University Zhuhai China

**Keywords:** age‐related macular degeneration, cataract, diabetic retinopathy, ophthalmic disease, sulforaphane

## Abstract

Sulforaphane (SFN) is an organosulfur compound categorized as an isothiocyanate (ITC), primarily extracted from cruciferous vegetables like broccoli and cabbage. The molecular formula of sulforaphane (SFN) is C_6_H_11_NOS_2_. SFN is generated by the hydrolysis of glucoraphanin (GRP) through the enzyme myrosinase, showing notable properties including anti‐diabetic, anti‐inflammatory, antimicrobial, anti‐angiogenic, and anticancer attributes. Ongoing clinical trials are investigating its potential in diseases such as cancer, neurodegenerative diseases, diabetes‐related complications, chronic kidney disease, cardiovascular disease, and liver diseases. Several animal carcinogenesis models and cell culture models have shown it to be a very effective chemopreventive agent, and the protective effects of SFN in ophthalmic diseases have been linked to multiple mechanisms. In murine models of diabetic retinopathy and age‐related macular degeneration, SFN delays retinal photoreceptor cell degeneration through the Nrf2 antioxidative pathway, NF‐κB pathway, AMPK pathway, and Txnip/mTOR pathway. In rabbit models of keratoconus and cataract, SFN has been shown to protect corneal and lens epithelial cells from oxidative stress injury by activating the Keap1‐Nrf2‐ARE pathway and the Nrf‐2/HO‐1 antioxidant pathway. Oral delivery or intraperitoneal injection at varying concentrations are the primary strategies for SFN intake in current preclinical studies. Challenges remain in the application of SFN in eye disorders due to its weak solubility in water and limited bioavailability because of the presence of blood–ocular barrier systems. This review comprehensively outlines recent research on SFN, elucidates its mechanisms of action, and discusses potential therapeutic benefits for eye disorders such as age‐related macular degeneration (AMD), diabetic retinopathy (DR), cataracts, and other ophthalmic diseases, while also indicating directions for future clinical research to achieve efficient SFN treatment for ophthalmic diseases.

## Graphical abstract

Sulforaphane (SFN) is an organosulfur compound categorized as an isothiocyanate (ITC), primarily extracted from cruciferous vegetables like broccoli and cabbage. This review comprehensively outlines recent research on SFN, elucidates its mechanisms of action, and discusses potential therapeutic benefits for eye disorders such as age‐related macular degeneration (AMD), diabetic retinopathy (DR), cataracts, and other ophthalmic diseases, while also indicating directions for future clinical research to achieve efficient SFN treatment for ophthalmic diseases.

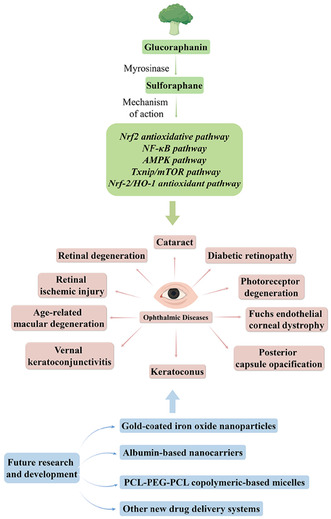

## 1 INTRODUCTION

**FIGURE 1:**
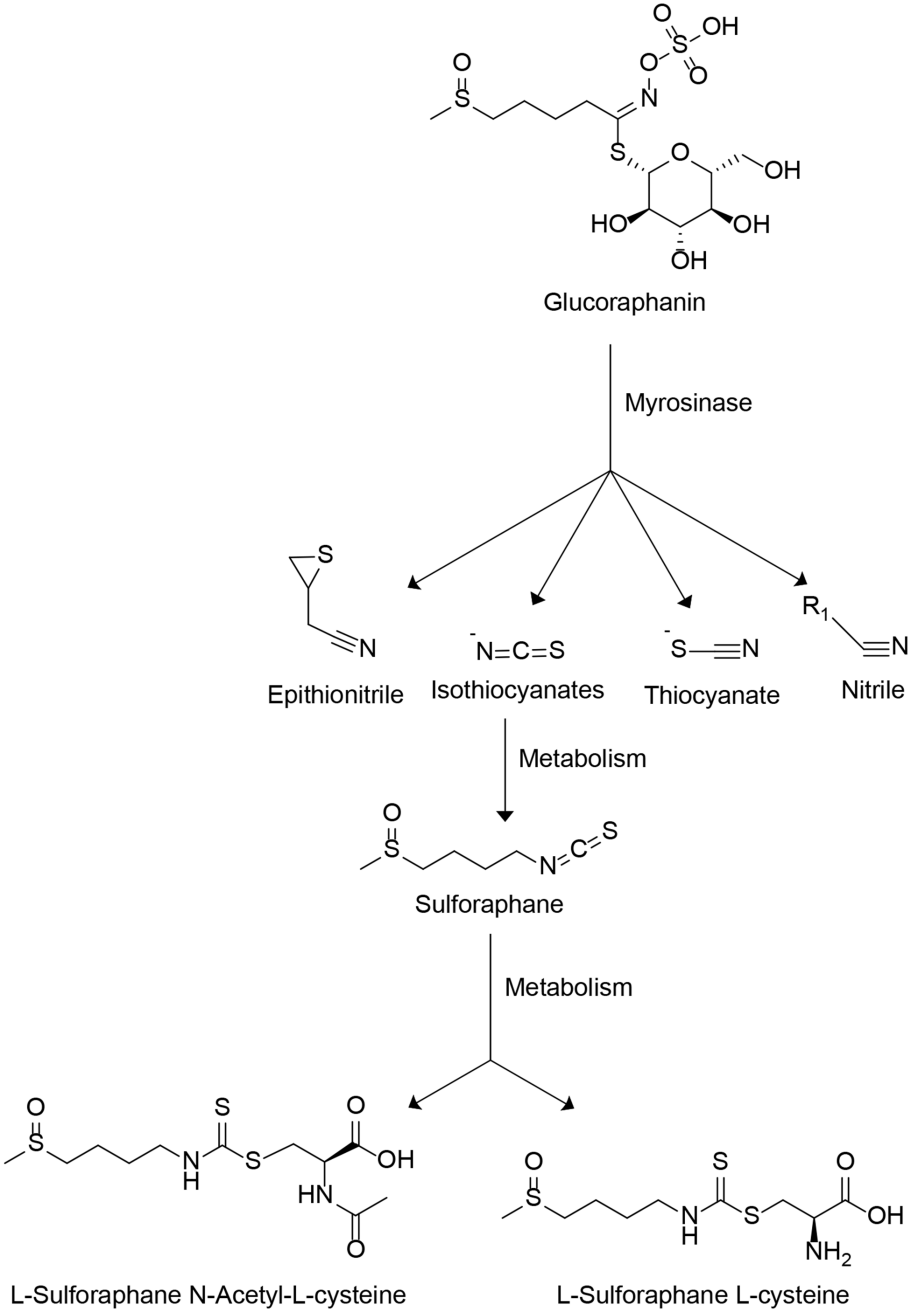
The metabolism of sulforaphane (SFN): with different environmental temperatures, PH, and coenzyme factors, the glucoraphanin (GRP) could be hydrolyzed by the myrosinase into epithionitrile, isothiocyanates, thiocyanate, and nitrile. As one of the most reactive degradation products, isothiocyanate (ITC) can transform into SFN, which will be further metabolized through the mercapturic acid pathway to synthesize LSF‐N‐Acetyl‐L‐cysteine (LSF‐NAC) and LSF‐L‐cysteine (LSF‐cys).

People worldwide are increasingly focusing on their vegetable consumption to maintain good health and prevent diseases. Growing evidence underscores the potential significance of cruciferous vegetables like broccoli and cabbage, known for their anti‐inflammatory and antioxidant characteristics, in addressing various disorders such as cancer, neurodegenerative diseases, diabetes‐related complications, chronic kidney disease, cardiovascular disease, liver diseases, and various eye diseases (Houghton, 2019; Liebman & Le, 2021; Liu et al., 2023; Mthembu et al., 2023; Russo et al., 2018; Schepici et al., 2020; Yan & Yan, 2023). Glucosinolates (GSLs), sulfur‐rich secondary metabolites primarily found as glucoraphanin (GRP), play a pivotal role in these cruciferous vegetables. GRP can be hydrolyzed by the enzyme myrosinase into various distinct compounds, including epithionitrile, nitrile, thiocyanate, and isothiocyanates (Bell et al., 2018; Halkier & Gershenzon, 2006). One of the most reactive degradation products, isothiocyanate (ITC), has the potential to transform into SFN. When plant tissues are damaged, such as through cutting, cytoplasmic GRP rapidly converts into specific ITCs through the vacuolar enzyme myrosinase (Vanduchova et al., 2019). Moreover, the human intestinal microbiota can break down GRP into specific ITCs, especially when myrosinase is inactivated after cooking (Figure 1) (Lai et al., 2010; Mazarakis et al., 2020; Sikorska‐Zimny & Beneduce, 2021; Yuanfeng et al., 2021).

This review comprehensively presents recent research on SFN, elucidates its mechanism of action, and discusses potential therapeutic benefits for eye disorders such as age‐related macular degeneration (AMD), diabetic retinopathy (DR), cataracts, and other ophthalmic diseases. Structured searches were conducted on prominent online databases, including PubMed, Embase, and Cochrane Library databases. Broad search terms were employed to identify relevant research, including sulforaphane, eye diseases, age‐related macular degeneration, diabetic retinopathy, and cataract. Extracted studies primarily focused on in vivo preclinical models of SFN. The subheadings in the review are listed as biological activity for SFN, mechanism of SFN, SFN and ophthalmic diseases, ongoing and completed clinical trials on SFN, safety and toxicity of SFN, future perspectives, and conclusion.

## 2 BIOLOGICAL ACTIVITY FOR SFN

SFN exhibits weak solubility in water but demonstrates solubility in organic solvents like methanol, ethanol, dimethyl sulfoxide, and ethyl acetate. It typically appears as a yellow or colorless liquid at room temperature. Moreover, the molecule is susceptible to disintegration at high temperatures, given its relatively low melting point of 74.6°C (Table 1). In human administration, SFN can be delivered either directly in its active form or as GRP. Upon oral administration, SFN is often absorbed from the intestine, displaying lower bioavailability, a shorter half‐life, and a significant first‐pass effect. Furthermore, SFN interacts with glutathione (GSH) and glutathione‐S‐transferase within cells, initiating the mercapturic acid pathway. Consequently, SFN conjugates with GSH to form LSF‐GSH, which undergoes further metabolism to produce LSF‐N‐Acetyl‐L‐cysteine (LSF‐NAC) and LSF‐L‐cysteine (LSF‐cys). Predominantly, SFN is excreted as LSF‐NAC, with approximately 70 to 75 percent excreted through urine (Clarke, Hsu, Williams, et al., 2011; Clarke, Hsu, Yu, et al., 2011; Conaway et al., 2000; Mazarakis et al., 2020). Notably, research by Okunade et al. (2018) revealed that incorporating brown mustard powder into cooked broccoli significantly enhances the bioavailability of SFN, potentially up to eightfold, consequently leading to a potential fourfold increase in the metabolite SFN‐NAC. Furthermore, clinical investigations have highlighted the influence of material sources on SFN bioavailability in the human body. For instance, peak plasma concentrations of SFN were seven times higher in subjects consuming fresh broccoli sprouts compared to broccoli supplements, with corresponding urine excretion five times higher. These findings underscore the substantially higher bioavailability of SFN when derived from whole food sources, facilitated by the presence of myrosinase in fresh broccoli sprouts (Clarke, Hsu, Riedl, et al., 2011; Fahey et al., 2015).

**TABLE 1:** Physicochemical properties of SFN.

Property name	Property value
Molecular formula	C_6_H_11_NOS_2_
Canonical SMILES	CS(=O)CCCCN=C=S
IUPAC name	1‐Isothiocyanato‐4‐methylsulfinylbutane
InChI	InChI = 1S/C6H11NOS2/c1‐10(8)5‐3‐2‐4‐7‐6‐9/h2‐5H2,1H3
Molecular weight	177.3 g/mol
UNII number	41684WL1GL
CAS number	4478‐93‐7
Physical description	Solid
Melting point	74.6°C
Boiling point	368.00–369.00°C

## 3 MECHANISM OF SFN

The ITC (‐N=C=S) group features an electrophilic carbon, imparting high activity to SFN compared to the biologically inert phytochemical protostar GRP. Recent research has elucidated SFN's potential as an anti‐diabetic, anti‐inflammatory, antimicrobial, anti‐angiogenic, anticancer, and antioxidant agent. The primary pharmacophore in SFN, the ITC group, is believed to induce cell apoptosis, phase G2/M cell cycle arrest, inhibition of phase‐I (carcinogen‐activating) enzyme, and stimulation of phase‐II (carcinogen‐detoxifying) enzyme. Moreover, SFN has the ability to scavenge free radicals and bind to a number of oxidizing agents, including hydroxyl, peroxide, and superoxide radicals. Previous research has demonstrated that the antioxidative function of SFN is associated with the activation of nuclear factor‐erythroid 2‐related factor 2 (Nrf2), a key regulator found in numerous organs (Zhou et al., 2022). Nrf2 is widely recognized as the “master redox switch” and “activator of cellular defense mechanisms”. Typically, Nrf2 resides in a complex with Kelch‐like ECH‐associated protein 1 (Keap‐1) and is tethered to cytosolic actin filaments. Upon detection of external stimuli by Keap‐1, Nrf2 dissociates from Keap‐1, translocating to the nucleus to upregulate the expression of target genes by binding to antioxidant response elements (AREs) in their upstream promoter regions (Dinkova‐Kostova et al., 2005; Houghton, 2019; Prestera et al., 1993). At the molecular level, SFN interacts with protein targets such as the mammalian target of rapamycin (mTOR), acting as both a potent Nrf2 activator and mTOR inhibitor, thus playing a critical role in cellular homeostasis regulation (Russo et al., 2018; Zhang et al., 2021). The properties of Nrf2 make it a promising novel drug target with potential applications across a wide range of conditions.

### 3.1 Anti‐diabetic activity

Diabetes mellitus (DM) is a chronic, noncommunicable disease that is characterized by hyperglycemia and insulin resistance. The progression of macrovascular, microvascular, and neuropathic changes in DM leads to complications such as kidney disease, blindness, and amputations, affecting over 8% of the global population (Tönnies et al., 2019). Current treatments for reducing glycemic levels primarily involve insulin injections and oral medications, both of which have the potential to decrease mortality and enhance the quality of life for diabetes patients. However, there are limited reports on DM prevention, and existing treatments simply focus on slowing down the disease's progression. Recent research by Wang et al. (2022) has demonstrated that SFN might activate Nrf2 to prevent diabetes‐induced oxidative stress and diabetic cardiomyopathy (Gu et al., 2017; Xin et al., 2018). Studies conducted by Li et al. (2020) have also indicated that SFN could prevent renal injury through Nrf2 antioxidative pathways and renal lipid metabolic pathways, both mediated by the 5′ adenosine monophosphate‐activated protein kinase (AMPK) signaling. Additionally, research by Wang et al. (2020) shown that SFN could boost muscle strength and prevent skeletal muscle dysfunction in people with spontaneous type 2 diabetes. SFN may also inhibit the expression of genes involved in glucose generation in hepatoma cells, leading to reduced glucose production. Moreover, in obese patients with dysregulated type 2 diabetes, highly concentrated SFN may significantly lower blood sugar levels, improving fasting glucose and glycated hemoglobin (Axelsson et al., 2017; Bahadoran et al., 2012). In summary, SFN would be an ideal choice for the treatment of type 2 diabetes.

### 3.2 Anti‐inflammatory activity

The nuclear factor kappa‐light‐chain‐enhancer of activated B cells (NF‐κB) and Nrf2 pathways serve as crucial mediators for SFN's anti‐inflammatory effects. During the inflammatory process, SFN effectively regulates mitogen‐activated protein kinase (MAPK) signaling, a key player in modulating both pro‐ and anti‐inflammatory responses in LPS‐activated microglia. Studies by Subedi et al. (2019) demonstrated significant reductions in MAPK phosphorylation levels in microglial cells following pre‐ and post‐treatment with SFN. On one hand, SFN diminishes c‐Jun N‐terminal kinase (JNK) phosphorylation levels, subsequently decreasing NF‐κB and activator protein‐1 (AP‐1) signaling. This reduction leads to decreased transcription of proinflammatory cytokines, including IL‐6, IL‐1β, and TNF‐α, as well as inflammatory mediators such as COX‐2, iNOS, PGE, and NO. On the other hand, SFN upregulates the expression of Nrf2 and heme oxygenase‐1 (HO‐1), potentially increasing the production of anti‐inflammatory cytokines like IL‐4 and IL‐10 (Subedi et al., 2019). Recent research by Yang et al. (2022) indicated that SFN can downregulate the expression of IL‐4Rα, TNFRI, and TNFRII. Consequently, decreased levels of phosphorylation were observed in MAPKs, STAT6, and IκBα, ultimately alleviating allergic inflammatory keratoconjunctivitis (Yang et al., 2022). Additionally, sulforaphane exhibits the potential to inhibit peritoneal cell recruitment and IL‐1β secretion in acute gout, reverse the resistance of *Bacillus anthracis* spore infection, and mitigate inflammatory injury to renal tubular epithelial cells. These properties position SFN as a promising treatment for chronic inflammatory diseases (Kim & Park, 2016; Liu et al., 2020; Mazarakis et al., 2020).

### 3.3 Antimicrobial activity

According to previous research on infectivity, SFN has shown promise in limiting the growth of bacterial pathogens, particularly *Helicobacter pylori*. SFN induces bacterial mortality by disrupting cell membrane integrity and inhibiting enzymes responsible for maintaining redox balance and bacterial metabolism (Romeo et al., 2018). This action of antibacterial has been specifically described. Moreover, research by Fahey et al. (2002) highlighted SFN's dual activities, indicating its potential to prevent *H. pylori* infections as well as the growth of stomach tumors. SFN demonstrates robust bacteriostatic activity against 3 reference strains and 45 clinical isolates of *H. pylori*. Around 90% of the strains exhibited satisfactory results, with a minimum inhibitory concentration (MIC) indicated as ≤4 μg/mL. Additionally, a brief exposure to SFN, such as following a cellular intake of 7.5 mol, can lead to bacterial death. SFN can also eliminate intracellular *H. pylori* from the human epithelial cell line (HEp‐2) (Fahey et al., 2002). Gram‐positive bacteria have been observed to be sensitive to SFN and its equivalents, as indicated by research by Cierpiał et al. (2020) SFN derivatives, particularly 16e, have demonstrated potent and effective action against *Staphylococcus aureus* strains, including methicillin‐resistant *S. aureus* (MRSA), providing a potential treatment option for antibiotic‐resistant strains. However, they have shown limited efficacy against gram‐negative bacteria such as *E. coli* and *P. aeruginosa*. Additionally, analogs with fluorophenyl substitution displayed superior antifungal efficacy compared to the parent SFN (Cierpiał et al., 2020). However, SFN tends to have specific effects within the human digestive tract. Abukhabta et al. (2021) reported that consuming broccoli soup enriched with SFN might suppress bacterial development in the human stomach and upper small intestine, but not in the terminal ileum or colon.

### 3.4 Anti‐angiogenic activity

Angiogenesis is a multifaceted process that includes the release of angiogenic factors, incorporation between angiogenic factors and endothelial cell receptors, activation, migration, and proliferation of endothelial cells (ECs), remodulation of the extracellular matrix, and the formation of tubes (Carmeliet & Jain, 2000). Angiogenic factors and interactions between endothelial cells and the perivascular matrix regulate angiogenesis. Nowadays, the anti‐angiogenic effect of SFN has been widely investigated. Nishikawa et al. (2010) conducted an in vitro model of angiogenic ECs by isolating human umbilical vein endothelial cells (HUVECs). They discovered that SFN could induce apoptosis, consequently inhibiting the proliferation ability of ECs. Moreover, it was found that combining SFN with an inhibitor of autophagy could enhance the effect on ECs (Nishikawa et al., 2010). To further explore the anti‐angiogenic potentials of SFN, Liu, Atkinson, et al. (2017) additionally used in vitro models involving HUVECs and human hepatocellular carcinoma cells (HepG2). Their findings indicated that SFN reduced the vitality, migration, and tube formation of HUVEC cells. Additionally, SFN significantly decreased HepG2‐induced HUVEC migration, adhesion, and tube formation by obstructing STAT3/HIF‐1α/VEGF signaling. However, a biphasic effect of SFN has been reported, where a high dose of SFN (>10 μM) demonstrated a significant anti‐angiogenic effect, while a low dose of SFN (2.5 μM) was found to promote cell migration and proliferation (Bao et al., 2014; He et al., 2021; Liu, Atkinson, et al., 2017; Zakaria et al., 2018). Moreover, Davis et al. (2009) demonstrated that SFN enhanced the inhibition of MEK/ERK and PI3K/AKT pathways, which can synergistically increase forkhead box O (FOXO) transcriptional activity; it can also inhibit cell migration and capillary tube formation. In conclusion, SFN inhibits angiogenesis through the activation of FOXO transcription factors and the inhibition of STAT3/HIF‐1α/VEGF signaling.

### 3.5 Anticancer activity

The anticancer properties of SFN have been extensively explored in recent years, demonstrating its potential to effectively inhibit the viability, proliferation, migration, malignancy, and epithelial‐to‐mesenchymal transition in cancer cells. SFN has shown its anti‐cancer actions in the skin, blood, breast, colon, prostate, and pancreas. Notably, SFN has been found to block stem cell formation and suppress the Wnt/β‐catenin pathway signaling, both of which are crucial in the treatment of triple‐negative breast cancer (Arzi et al., 2022; Li et al., 2010). In a recent study by Zhang et al. (2022), SFN and its two isomers, R‐SFN and S‐SFN, significantly reduced migration and invasion induced by TGF‐1 in breast cancer cells. They also reported that SFN inhibited the formation of actin stress fibers by downregulating RAF/MEK/ERK pathway signaling, ultimately reducing breast cancer cell metastasis (Zhang et al., 2022). Additionally, Aumeeruddy and Mahomoodally (2019) observed that a combination of three phytochemicals—SFN, piperine, and thymoquinone—could enhance therapeutic efficacy against breast cancer compared to individual treatments. SFN has been observed to induce apoptosis in hepatocellular carcinoma cells, causing morphological changes such as cell contraction, blistering, chromatin condensation, and nuclear fragmentation (Wu et al., 2020). Furthermore, SFN reduced the viability and telomerase activity of hepatocellular carcinoma Hep3B cells by activating ROS‐dependent pathway signaling and reducing microtubule polymerization (Moon et al., 2010; Pocasap et al., 2018). Research by Ren et al. (2017) indicated that SFN improved the radiosensitivity of hepatocellular carcinoma by inhibiting NF‐κB pathway signaling, which plays an essential role in the development of liver cancer. They also found that, SFN suppressed the expression of downstream genes in the NF‐kB pathway in hepatocellular carcinoma cells (Ren et al., 2017). In addition, the molecular pathway of antitumor activity has also been reported as a novel therapy target (Rafiei et al., 2020). Rafiei et al. (2020) discovered that SFN has excellent miRNA modulatory capability, with the ability to either up‐ or down‐regulate miRNA expression in the promotion or repression of cancer.

### 3.6 Anti‐oxidant activity

As an essential factor in the antioxidant defense system, the expression of Nrf2 significantly increased after SFN treatment, which could activate cellular antioxidant enzymes and protect against oxidative stress. The Nrf2‐ARE signal pathway plays a pivotal role in upregulating protective genes and proteins associated with antioxidant properties, encompassing both direct antioxidant enzymes (like catalase, superoxide dismutase, and GSH peroxidase) and indirect antioxidant enzymes (like GSH generation enzymes and Phase‐2 detoxification enzymes) (de Figueiredo et al., 2015). In addition, research conducted by Lv, Meng, et al. (2020) revealed that the antioxidative properties and SFN levels of broccoli vary among cultivars, seeds, and sprouts. Interestingly, they found that broccoli sprouts exhibited greater antioxidant capability than the original seeds, despite lower SFN levels in the sprouts compared to the seeds (Table 2) (Lv, Meng, et al., 2020).

**TABLE 2:** Biological activity and mechanism of action of SFN.

Biological activity	Effect	Mechanism of action	Reference
Anti‐diabetic	Reduce glucose production, prevent diabetes‐induced cardiomyopathy, renal injury, and skeletal muscle dysfunction	AMPK mediated activation of Nrf2 antioxidative and renal lipid metabolic pathways	Li et al. (2020)
Anti‐inflammatory	Reduce levels of inflammatory mediators and proinflammatory cytokines	Upregulate Nrf2 and HO‐1 pathways; downregulate NF‐κB, MAPK, STAT6, and AP‐1 pathways	Subedi et al. (2019) and Yang et al. (2022)
Antimicrobial	Inhibit bacterial pathogen growth, reduce bacterial infections, kill bacteria, and block gastric tumor formation	Destroy cell membrane integrity, inhibit enzymes involved in redox balance and bacteria metabolism	Romeo et al. (2018)
Anti‐angiogenic	Inhibit proliferative ability, cell viability, migration, adhesion, and tube formation	Induct apoptosis, activate FOXO transcription factors, and inhibit STAT3/HIF‐1α/VEGF signaling	Carmeliet and Jain (2000) and Davis et al. (2009)
Anticancer	Promote apoptosis, inhibit cell viability, proliferation, metastasis, malignancy, and epithelial‐to‐mesenchymal transition	Downregulate Wnt/β‐catenin, RAF/MEK/ERK, and NF‐κB pathways	Arzi et al. (2022), Li et al. (2010) and Zhang et al. (2022)
Anti‐oxidant	Activate cellular antioxidant enzymes	Upregulate Nrf2‐ARE signal pathway	de Figueiredo et al. (2015)

## 4 SFN AND OPHTHALMIC DISEASES

### 4.1 Age‐related macular degeneration

According to the World Health Organization, AMD has been listed in the top 10 eye diseases and has become a leading cause of blindness among elderly people. AMD, a degenerative macular retinal disease, severely impairs vision in affected elderly adults. While various risk factors contribute to AMD, oxidative stress and choroidal vascular dysfunction play critical roles in its pathogenesis (Kwa et al., 2019; Ruan et al., 2021). Current treatments primarily focus on vascular endothelial growth factor inhibitors, and SFN has emerged as a promising treatment for AMD. The model of mouse retinal degeneration has been established and applied to many clinical trials. Age‐related changes in Nrf2 expression and function are observed in retinal pigment epithelial (RPE) cells of old mice (Sachdeva et al., 2014). Qi et al. (2022) evaluated whether the lack of the transcription factor constrains the therapeutic efficacy of SFN against retinal degeneration. They suggested that SFN could retain the function of the cone, but in mice with reduced Nrf2 function, the efficiency may be limited (Qi et al., 2022). The pathophysiology of inflammatory injury to RPE cells has been investigated using microarray analysis. Ye et al. (2013) indicated that, after treatment with SFN, significant changes were observed in the transcription of 69 genes in human retinal pigment epithelium 19 (ARPE‐19) cells. Several processes, including anti‐apoptosis, anti‐oxidation, and cell growth regulation, were associated with these cells. SFN improved the antioxidative ability of RPE 19 cells by upregulating antioxidative genes (including quinone oxidoreductase [NQO1], sulfiredoxin1 homolog [SRXN1], thioredoxin 1 [Trx1], and glutamate‐cysteine ligase modifier subunit [GCLM]), and downregulating inflammatory response genes (such as thioredoxin interacting protein [TXNIP], chemokine [C‐C motif] ligand 2 [CCL2], and bradykinin receptor B1 [BDKRB1]). Moreover, through Nrf2 antioxidative pathways, SFN may modulate the promoters of the antioxidant response elements (NQO1, GCLM, and Trx1) (Ye et al., 2013). Furthermore, Song et al. (2022) showed that SFN prevents lipopolysaccharide (LPS)‐induced inflammatory damage to RPE cells by inhibiting the PWRN2/NF‐kB pathway. They suggested that SFN blocked NF‐kB activation and downregulated PWRN2 in a concentration‐dependent manner. Conversely, NF‐kB upregulation or PWRN2 overexpression reduced the anti‐inflammatory effects of SFN (Song et al., 2022). The RPE cell layer plays an important role in protecting retinal photoreceptors from oxidative stress. Furthermore, it has been indicated that declining protective capacity may play a significant role in AMD development. By enhancing Trx expression in the mouse retina, Tanito et al. (2005) demonstrated that SFN could reduce retinal light damage. Additionally, intraperitoneal and oral administration of SFN upregulates the expression of the Trx gene in RPE cells by antioxidant response element (ARE) and affords cytoprotection against light‐induced RPE and photoreceptor cell damage in mice. Although SFN plays an indirect role in antioxidants, it could induce the transcription of phase II genes, thereby exerting the antioxidant effect. By promoting the expression of Trx and phase II enzymes, SFN may be a useful preventative measure for retinal illnesses caused by photooxidative stress, which may worsen the severity and progression of AMD (Tanito et al., 2005). Gao and Talalay (2004) also demonstrated that SFN prevented photooxidative damage to RPE cells by promoting the expression of phase 2 genes. Pre‐conditioning RPE cells with SFN, acting as an inducer of phase 2 genes, demonstrated a considerable level of protection. The degree of protection was consistent with the capacity of inducers to enhance cytoprotective GSH levels and NAD(P)H activities (Gao & Talalay, 2004). Moreover, polyunsaturated fatty acids have been shown to increase the incidence of AMD, while oxidized lipids may contribute to the innate immunological dysfunction associated with oxidative stress in AMD. According to Kwa et al. (2019), L‐sulforaphane (LSF) can protect adult pigment epithelial cells against oxidative damage by upregulating the gene expression of Glutathione‐S‐Transferase μ1 enzyme. They also indicated that retinal cells faced with oxidative damage and apoptosis risks should be pre‐conditioned with LSF, which could retard AMD progression by regulating fatty acids and lipids involved in downstream pathways (Kwa et al., 2019). Recent research by Sim et al. (2021) discovered the molecular basis of SNF's protective properties against particulate matter 2.5 (PM2.5)‐induced toxic damage in ARPE‐19 cells. Increased intracellular reactive oxygen species (ROS) levels or decreased antioxidant enzyme activity may result from exposure to PM2.5. Regarding AMD, the chronically excessive production and accumulation of ROS in RPE cells may be the primary cause of photoreceptor loss in the ultimate stage (Mao et al., 2014). However, SFN may be able to reduce the oxidative stress caused by PM2.5 and subsequently increase the viability of ARPE‐19 cells. Meanwhile, the pre‐treatment with SNF could significantly reverse the pro‐apoptotic changes, such as reduced protein levels of Bax, cleaved caspase‐3, as well as cytosolic cytochrome c, and increased levels of Bcl‐2 (Sim et al., 2021). Taken together, these results suggest that the antioxidant SFN, especially when exposed to PM2.5, may be a valuable and potential therapeutic agent for AMD.

### 4.2 Diabetic retinopathy

Diabetic retinopathy (DR), one of the most severe complications of diabetes, has emerged as a leading cause of blindness, significantly impacting the quality of life for many diabetic patients (Antonetti et al., 2012). An early histopathologic change associated with DR is the loss of pericytes, often occurring without noticeable symptoms. However, once visual symptoms manifest, the progression of DR can become irreversible and potentially lead to blindness (Ting et al., 2016). Several distinct changes characterize DR, including the development of microaneurysms, thickening of the retinal basement membrane, and increased permeability of the retinal vessels. These changes are attributed to oxidative stress, the accumulation of advanced glycation end products (AGEs), inflammasome activation, and inflammation (Lai et al., 2017; Shruthi et al., 2017; Tönnies et al., 2019). Research by Lv, Bao, et al. (2020) demonstrated that SFN could retard retinal photoreceptor cell degeneration in DR. SFN was found to delay the thinning of the ganglion cell layer, inner nuclear layer, outer nuclear layer, and total retina, ultimately reducing photoreceptor loss. Additionally, SFN was observed to reduce AGEs‐induced damage in 661w cells by activating the AMPK pathway, subsequently reducing the expression of 78 kDa glucose‐regulated protein (GRP78), tumor necrosis factor‐α (TNFα), and Txnip (Lv, Bao, et al., 2020). Li et al. (2019) proposed that SFN protects against DR by activating the Nrf2 pathway and inhibiting the formation of the NLRP3 inflammasome. Furthermore, SFN was shown to decrease TNF‐, IL‐1, and IL‐6 levels, improve mRNA and protein levels, enhance antioxidant activity, and downregulate the expression of inflammasome components (Li et al., 2019). It is widely recognized that AGEs can trigger excessive autophagy, potentially resulting in cell death and exacerbating the course of DR as AGEs accumulate (Chawla et al., 2014). Ren et al. (2018) discovered that mTOR could lead to AGE accumulation in diabetic mice, inducing retinal degeneration by downregulating Trx and upregulating Txnip signaling. In contrast, SFN, a potent mTOR inhibitor, could upregulate Trx, resulting in the increase in retinal thickness observed in diabetic mice. Furthermore, SFN, by inhibiting mTOR and upregulating Trx, may inhibit autophagy through the Txnip/mTOR pathway, thereby retarding neurodegeneration induced by AGEs (Ren et al., 2018). AGEs can also trigger inflammation and apoptosis in retinal pericytes through interaction with AGE receptors. Therefore, modulating the AGE‐RAGE axis using SFN may present a promising therapeutic target for treating DR (Maeda et al., 2014; Yamagishi & Matsui, 2016).

### 4.3 Cataract

Cataract, characterized by lens opacification, is effectively treated through surgical intervention, leading to rapid recovery post‐surgery (Asbell et al., 2005). However, approximately 20% of patients may experience complications known as posterior capsule opacification (PCO), resulting from residual lens epithelial cell adhesion to the anterior capsule after surgery. PCO is typically treated with a neodymium:YAG laser, but this approach carries some complications and risks (Wormstone et al., 2021). Recent research has revealed that SFN exhibits a hormetic effect, contributing to both cytotoxicity and cytoprotection. On the one hand, SFN is believed to have cytotoxic actions. Once the concentrations reach 10 μM and above, they may significantly interfere with lens cell wound healing, which could eventually prevent PCO. Huynh et al. (2021), using models of the central anterior epithelium and the human lens epithelial cell line FHL124, found that SFN depletes GSH in lens cells by downregulating GSH reductase activity. This accumulation of oxidative stress and ROS promotes progression, including endoplasmic reticulum stress (ERS), mitochondrial dysfunction, autophagy, and DNA damage, ultimately resulting in lens cell death (Huynh et al., 2021). Liu, Smith, et al. (2017) also suggested that SFN could retard the growth, migration, and viability of lens epithelial cells. Concurrently, SFN‐induced autophagy was regulated by MAPK signaling and MEK signaling (Liu, Smith, et al., 2017). On the other hand, SFN has been reported to upregulate the activity of Nrf2/ARE/Prdx6 peroxiredoxin and activate cellular antioxidant enzymes, thereby protecting aging lens epithelial cells. Additionally, SFN enhances the survivability of lens epithelial cells and reactivates Nrf2 in aged and dysregulated cells (Calabrese & Kozumbo, 2021). In lens epithelial cells exposed to hydrogen peroxide, Liu et al. (2013) found that SFN could reduce DNA damage, transparency loss, and cell death. They also demonstrated that SFN could protect lens cells from oxidative stress by activating Keap1‐Nrf2‐ARE pathway (Liu et al., 2013). Furthermore, Varma et al. (2013) suggested that SFN increased the transcription of thioredoxin reductase in lens cells, eventually reducing oxidative stress. In conclusion, SFN exhibits a concentration threshold for its opposing actions, with the level determining cell viability and death. In lens epithelial cells, SFN demonstrates cytoprotective effects at lower concentrations, making it a potential tool for repairing and reversing cataracts. In contrary, at concentrations of 10 μM and above, SFN displays cytotoxicity and may serve as a potential therapy for PCO.

### 4.4 Other eye‐related diseases

Several studies also reported the promising role of SFN in other eye‐related diseases. In vernal keratoconjunctivitis (VKC), Yang et al. (2022) demonstrated that SFN possesses anti‐inflammatory and anti‐allergenic characteristics. SFN was found to inhibit the expression of chemokines and adhesion molecules triggered by the co‐stimulation of TNF‐α and IL‐4 in human corneal fibroblasts. These included vascular cell adhesion molecule‐1, thymus‐ and activation‐regulated chemokines, and eotaxin‐1. The inhibitory effect was likely mediated by downregulating the expression of IL‐4Rα, TNFRI, and TNFRII, and reducing phosphorylation levels in MAPKs, IκBα, and STAT6. Hence, SFN holds potential as a candidate for treating VKC due to its efficacy in suppressing late‐phase allergic inflammation (Yang et al., 2022). In the study of keratoconus, Liu and Yan (2018) found that SFN, through activation of the Nrf‐2/HO‐1 antioxidant pathway, could protect rabbit corneas from oxidative stress. This protection led to lowered keratometry and increased central cornea thickness, ultimately inhibiting the progression of keratoconus (Liu & Yan, 2018). Moreover, Kang and Yu (2017) suggested that continuous treatment with SFN could significantly inhibit GRP78/BiP expression in the ER of the rd10 retina, indicating that SFN could reduce ERS and subsequently reduce photoreceptor apoptosis. This protective effect demonstrated in a mouse model of retinitis pigmentosa highlights SFN as a potential drug to ameliorate retinal degeneration (Kang & Yu, 2017). Ambrecht et al. (2015) initially reported the protective effect of SFN on retinal ischemic injury. SFN was shown to significantly alleviate ischemia‐induced retinal dysfunction and the inner retinal layers' attenuation compared to vehicle‐treated mice. Besides, they demonstrated that SFN therapy given at a dose of 25 mg/kg/day for 5 days can have neuroprotective effects on both the initial ischemia injury and the ensuing reperfusion injury. The neuroprotective effects were attributed to the activation of the antioxidant Nrf2/HO‐1 pathway. SFN could be a potential treatment for diseases causing retinal ischemia, such as diabetic retinopathy and retinal vascular occlusions (Ambrecht et al., 2015; Pan et al., 2014). In Fuchs endothelial corneal dystrophy (FECD), Ziaei et al. (2013) demonstrated the effectiveness of SFN. By enhancing Nrf2‐ARE pathway signaling, SFN was able to reduce corneal endothelial cell apoptosis in FECD (Ziaei et al., 2013). Additionally, studies by Kong et al. (2007) showed that SFN could retard photoreceptor degeneration in tubby mice. Intraperitoneal injection with SFN significantly elevated Nrf2, Trx, and TrxR levels in the retina, providing protection to photoreceptor cells in tubby mice and potentially preventing inherited neurological disorders, including retinal dystrophic syndromes and retinitis pigmentosa (Table 3) (Kong et al., 2007).

**TABLE 3:** The effects and mechanism of action of SFN in ophthalmic diseases treatment.

Ophthalmic diseases	Effect	Mechanism of action	Reference
AMD	Improve the antioxidative ability of RPE 19 cells, reduce retinal light damage in RPE and photoreceptor cells, and reverse pro‐apoptotic changes	Increase the expression of antioxidative genes, Trx and phase II enzymes, downregulate inflammatory response genes, decrease oxidative stress	Song et al. (2022), Tanito et al. (2005) and Ye et al. (2013)
DR	Retard autophagy and neurodegeneration in retinal photoreceptor cells	Reduce oxidative stress, AGE accumulation, inflammasome activation, and inflammation	Lai et al. (2017), Ren et al. (2018) and Shruthi et al. (2017)
Cataract	Intervene wound‐healing in lens cells and protect aging lens epithelial cells.	Downregulate glutathione reductase activity, upregulate the activity of Nrf2/ARE/Prdx6 peroxiredoxin, and activate cellular antioxidant enzymes	Calabrese and Kozumbo (2021) and Huynh et al. (2021)
VKC	Inhibit expressions of chemokine and adhesion molecules in human corneal fibroblasts	Reduce the phosphorylation levels of MAPKs, IκBα, and STAT6	Yang et al. (2022)
Keratoconus	Protects corneas against oxidative stress injury	Activate the Nrf‐2/HO‐1 antioxidant pathway	Liu and Yan (2018)
Retinitis pigmentosa	Reduce photoreceptor apoptosis and retinal degeneration	Inhibit GRP78/BiP expression	Kang and Yu (2017)
Retinal ischemic injury	Alleviated ischemic‐induced retinal dysfunction and inner retinal layer attenuation	Activate the Nrf2/HO‐1 antioxidant pathway	Ambrecht et al. (2015) and Pan et al. (2014)
FECD	Reduce the apoptosis of corneal endothelial cells	Upregulate the Nrf2‐ARE pathway	Ziaei et al. (2013)
Retinal dystrophic syndromes	Retard photoreceptor degeneration	Increase levels of Nrf2, Trx, and TrxR	Kong et al. (2007)

Abbreviations: AGEs, advanced glycation end products; AMD, age‐related macular degeneration; DR, diabetic retinopathy; FECD, fuchs endothelial corneal dystrophy; RPE‐19 cells, retinal pigment epithelium 19 cells; SFN, sulforaphane; VKC, vernal keratoconjunctivitis.

## 5 ONGOING AND COMPLETED CLINICAL TRIALS ON SFN

Prior to its introduction to the market, extensive clinical trials were conducted to ensure the safety and efficacy of herbal medicines containing SFN. Table 4 provides a comprehensive overview of recent preclinical evidence concerning the therapeutic effects of SFN, sourced from the official clinical trial government website. SFN has been utilized as a dietary supplement for various disorders, including autism spectrum disorder, schizophrenia, lung cancer, prostate cancer, and chronic kidney disease. The majority of these trials have demonstrated considerable efficacy with SFN. Notably, a randomized, placebo‐controlled, multidose trial was designed to evaluate SFN's efficacy in mitigating the long‐term health risks associated with air pollution. The results revealed that SFN exhibited the ability to enhance the detoxification of the airborne pollutant benzene (Chen et al., 2019; Johns Hopkins Bloomberg School of Public Health et al., 2016). Additionally, clinical trials investigating SFN's effects on children with autism spectrum disorder have explored its efficacy, safety, and tolerability. Significant changes in biomarkers, including mitochondrial respiration, GSH redox status, inflammatory markers, and heat shock proteins, were observed in SFN groups compared to placebo groups (University of Massachusetts, Worcester et al., 2015; Zimmerman et al., 2021). Furthermore, observational studies have examined SFN's role as an adjunctive treatment for schizophrenia patients who were unresponsive to antipsychotic treatment (Central South University et al., 2018; Xiao et al., 2021). Despite the significant efficacy demonstrated in numerous clinical trials, further high‐quality research is necessary to conclusively demonstrate SFN's clinical therapeutic efficacy. Continued research efforts will contribute to a deeper understanding of SFN's potential benefits and ensure its safe and effective utilization in clinical practice.

**TABLE 4:** Recent clinical trials of SFN (ongoing and completed).

NCT number	Disease	Study type	Phases	Study design	Age	Enrollment	Interventions	Study status	Reference
NCT02909959	Autism spectrum disorder	Interventional	Phase 2	Randomized	13–30	48	Avmacol® 3–8 tablets orally daily, each tablet providing approximately 15 μmol SFN	May 2019 (Completed)	University of North Carolina, Chapel Hill and North Carolina Translational and Clinical Sciences Institute (2017)
NCT02656420	Environmental carcinogenesis	Interventional	Phase 1 Phase 2	Randomized	21–65	170	Maximum, half and one‐fifth doses of broccoli sprout‐derived beverage	March 2016 (Completed)	Johns Hopkins Bloomberg School of Public Health et al. (2016)
NCT02810964	Schizophrenia	Interventional	Phase 2	Randomized	18–65	64	Avmacol® 6 tablets orally daily	November 2019 (Completed)	Sheppard Pratt Health System (2017)
NCT02561481	Autism spectrum disorder	Interventional	Phase 1 Phase 2	Randomized	3–12	60	Tablet will be administered once a day orally containing broccoli seed powder equivalent to 45–120 μmol SFN	January 2020 (Completed)	University of Massachusetts, Worcester et al. (2015)
NCT03232138	Lung cancer	Interventional	Phase 2	Randomized	55–75	67	SFN four tablets 2 times per day with breakfast and dinner; each dose contains approximately 120 μmol of SFN	October 2023 (Ongoing)	Yuan et al. (2018)
NCT02677051	Autism	Interventional	Phase 2	Randomized	13–30	48	Oral pills containing broccoli seed powder equal to 50–150 μmol SFN for 22 weeks	July 2024 (Ongoing)	The State University of New Jersey Rutgers and Rowan University (2016)
NCT03665922	Prostate cancer	Interventional	NA	Randomized	18–90	39	Oral four BroccoMax® tablets with breakfast and four tablets with dinner. The eight BroccoMax® tablets will provide a daily internal dose of 64 mg of SFN	December 2024 (Ongoing)	University of Pittsburgh and National Cancer Institute (2019)
NCT02614742	Subarachnoid hemorrhage, spontaneous	Interventional	Phase 2	Randomized	18–80	90	Sulforadex® 300 mg bid for up to 28 days	November 2019 (Completed)	Evgen Pharma (2016)
NCT05797506	Chronic kidney disease stage 3–4	Interventional	Phase 2	Randomized	18–80	100	Oral 4 tablets of SFN (Avmacol Extra Strength) per day in patients with chronic kidney disease, stages 3–4	December 2025 (Ongoing)	University of Rochester et al. (2023)
NCT05408559	Diastolic dysfunction	Interventional	Phase 1 Phase 2	Randomized	60–80	200	Oral 2–4 caplets containing SFN‐rich broccoli sprout extracts (Avmacol Extra Strength) for 24 weeks	July 2026 (Ongoing)	Texas Tech University Health Sciences Center (2022)
NCT03932136	Clinical high risk syndrome of psychosis	Interventional	Phase 3	Randomized	15–45	300	Oral 6 tablets containing SFN per day for 52 weeks	December 2026 (Ongoing)	Shanghai Jiao Tong University School of Medicine et al. (2019)

## 6 SAFETY AND TOXICITY OF SFN

In vitro, in vivo, and clinical trials have extensively investigated the toxicological and biological effects of oral broccoli sprouts and SFN, revealing these plant‐derived bioactive compounds to be generally safe with beneficial biological effects. In vitro studies have demonstrated that while SFN exhibits cytotoxicity and anticancer activity in cancer cells, it poses minimal cytotoxicity to normal cells, enhancing its safety profile. Furthermore, clinical trials have corroborated the safety of SFN, with no severe adverse effects reported in individuals receiving the medication for short durations, ranging from days to weeks. The administration of SFN at proper dosages is crucial, with research indicating that the mean effective oral dose is 175 μmol/kg body weight and 113 μmol/kg when administered intraperitoneally. SFN has been deemed generally safe at and below these reported levels (Mangla et al., 2020). A few modest adverse effects, such as constipation, bloating, nausea, and headaches, have been reported in some patients (Alumkal et al., 2015; Zhang et al., 2020). Remarkably, even in children aged 3–12 years, as confirmed by a 15‐week randomized parallel double‐blind placebo‐controlled clinical laboratory study, SFN demonstrated favorable treatment tolerance, with few apparent side effects noted apart from rare instances of insomnia, irritability, and intolerance of taste and smell (University of Massachusetts, Worcester et al., 2015; Zimmerman et al., 2021). Moreover, pre‐clinical experiments have indicated that SFN can potentiate the anticancer activity of drugs such as doxorubicin, tamoxifen, and cisplatin while mitigating off‐target toxicity through multiple mechanisms (Calcabrini et al., 2020). For example, SFN could mitigate doxorubicin‐induced cardiotoxicity by enhancing mitochondrial functions in the heart and alleviate cisplatin‐induced nephrotoxicity by preventing changes in mitochondrial functionality in the kidney (Guerrero‐Beltrán et al., 2010; Kerr et al., 2018).

## 7 FUTURE PERSPECTIVES

In recent years, SFN has emerged as a promising phytochemical for the treatment of several diseases, including cancer, neurodegenerative diseases, diabetes‐related complications, chronic kidney disease, cardiovascular disease, and liver diseases. Recent in vitro and in vivo experimental studies demonstrated that SFN may have treatment effects in AMD, DR, cataracts, and other ophthalmic diseases. Despite these encouraging preclinical findings, SFN has yet to be clinically tested. Therefore, advanced research and clinical trials investigating SFN's efficacy in treating eye diseases are warranted in the future. However, there are three main issues that require resolution in future research.

Firstly, enhancing SFN stability through formulation modifications is a critical direction for future studies. SFN exhibits weak solubility in water and displays lower bioavailability, a shorter half‐life, and a significant first‐pass effect, which limit its development. To enhance efficacy in treating ocular diseases, future development efforts may concentrate on selecting appropriate carrier materials, exploring suitable surfactant types and dosages, and determining the optimal drug‐to‐lipid ratio.

Secondly, delivering drugs to the eye through blood–ocular barrier systems remains a major challenge. While systemic medication administration is one approach to treating ocular diseases, drug transfer to the retina via circulating blood is primarily regulated by two blood–ocular barrier systems: the blood–retinal barrier and the blood–aqueous barrier (Tomi & Hosoya, 2010). Thus far, only a few safe and efficient drug delivery systems have been reported, with nanotechnological formulations being commonly employed. Gold‐coated iron oxide nanoparticles, albumin‐based nanocarriers, and PCL‐PEG‐PCL copolymeric‐based micelles have been explored to enhance SFN bioavailability in the eye (Kheiri Manjili et al., 2016, 2017; Naqvi et al., 2022). Further understanding of drug transporters expressed in the blood–retinal barrier and corneal epithelium may facilitate the development of more efficient medication delivery systems for ocular diseases (Jordán & Ruíz‐Moreno, 2013). Moreover, as drug delivery systems advance, novel carriers capable of crossing blood–ocular barrier systems may be employed to treat eye conditions such as keratitis, AMD, and DR.

Thirdly, optimizing the dose of SFN intake is imperative for future research. Previous clinical trials have typically administered SFN‐containing intervention drugs daily with a single dose. However, SFN's short elimination half‐life in plasma, due to rapid metabolism by phase I and phase II drug metabolism enzymes, may restrict further absorption and distribution in the human body (Clarke, Hsu, Williams, et al., 2011). Consequently, the effective dose range of SFN remains unknown, with few clinical trials reporting dose responses to the drugs. Moreover, the doses used in the majority of animal experiments have exceeded the maximum dosage of SFN administered to humans. Thus, comprehensive dose–response studies of SFN are necessary to provide crucial information for establishing sensible SFN dose regimens to improve safety and efficiency in clinical translation.

## 8 CONCLUSION

**FIGURE 2:**
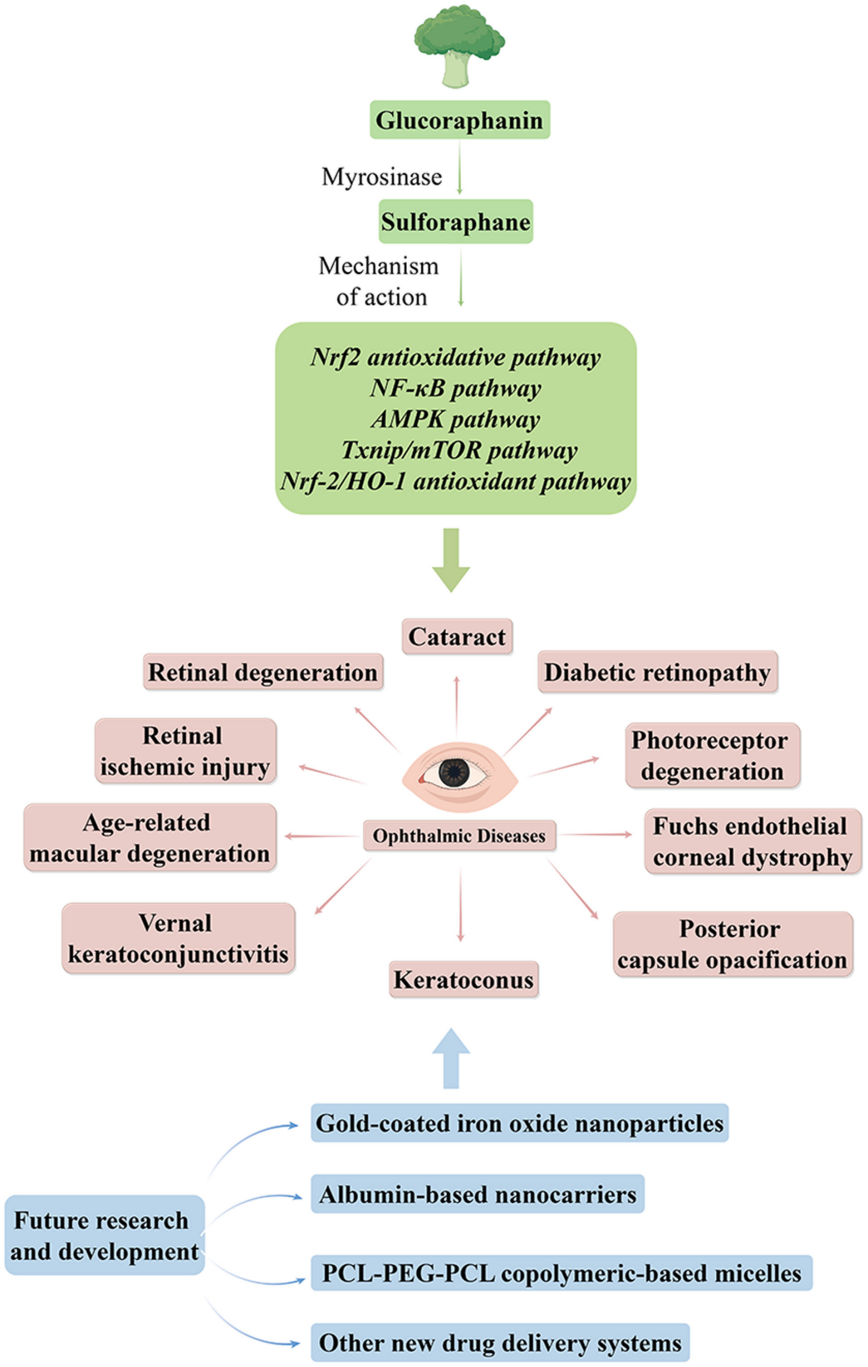
The mechanism and future development of SFN for eye diseases [By Figdraw.]: SFN can be applied in treating various ophthalmic diseases like AMD, DR, cataract, PCO, VKC, keratoconus, retinal degeneration, retinal ischemic injury, FECD, and photoreceptor degeneration. The protective effects of SFN have been linked to mechanisms including the Nrf2 antioxidative pathway, NF‐κB pathway, AMPK pathway, Txnip/mTOR pathway, and Nrf‐2/HO‐1 antioxidant pathway.

In conclusion, other than anti‐diabetic, anti‐inflammatory, antimicrobial, anti‐angiogenic, anticancer, and antioxidant activities, SFN can be used in ophthalmic diseases such as AMD, DR, cataract, PCO, VKC, keratoconus, retinal degeneration, retinal ischemic injury, FECD, and photoreceptor degeneration. Among them, a number of signal pathways, such as the Nrf2 antioxidative pathway, NF‐κB pathway, AMPK pathway, Txnip/mTOR pathway, Keap1‐Nrf2‐ARE pathway, and Nrf‐2/HO‐1 antioxidant pathway, present significant protective effects. There are several limitations, such as poor solubility in water, low bioavailability, and the formidable blood–ocular barrier systems, SFN has yet to be clinically developed for the treatment of eye disorders. Nonetheless, owing to its numerous health benefits and advancements in novel drug delivery systems, the clinical application and promotion of SFN in ocular diseases hold promise for the future. Upon successful completion of rigorous clinical trials, future endeavors may focus on developing novel drug delivery systems tailored for ocular applications. Such advancements hold the potential to greatly facilitate the clinical translation of SFN, paving the way for its broader therapeutic use in ocular diseases (Figure 2).

## AUTHOR CONTRIBUTIONS

**Yichi Zhang:** Data curation (equal); investigation (equal); writing – original draft (equal). **Xiaojing Zhao:** Data curation (equal); investigation (equal); writing – original draft (equal). **Yang Liu:** Supervision (equal); writing – review and editing (equal). **Xiuxia Yang:** Supervision (equal); writing – review and editing (equal).

## CONFLICT OF INTEREST STATEMENT

The authors declare no conflicts of interest.

## ETHICS STATEMENT

This study does not involve any human or animal testing.

## INFORMED CONSENT

Written informed consent was obtained from all study participants.

## Supporting information


Data S1

## Data Availability

Data sharing is not applicable to this article as no new data were created or analyzed in this study.
