# Supplementary material for: Sulforaphane and ophthalmic diseases
Source: Food Sci Nutr. 2024 May 22;12(8):5296–311. doi: 10.1002/fsn3.4230 (PMC11317731; doi:10.1002/fsn3.4230)
Supplement: Supplementary file 1 — Data S1 [file FSN3-12-5296-s001.docx]

**Sulforaphane and Ophthalmic Diseases**

***ONLINE SUPPLEMENT***

**Supplemental Figure 1：**The metabolism of sulforaphane (SFN).

**Supplemental Figure 2：**The mechanism and future development of SFN for eye diseases.

**Supplemental Table-1：**Physicochemical properties of SFN.

**Supplemental Table-2：**Biological activity and mechanism of action of SFN.

**Supplemental Table****-3：**The effects and mechanism of action of SFN in ophthalmic diseases treatment.

**Supplemental Table-4：**Recent clinical trials of SFN (ongoing and completed).

**Supplemental** **Figure 1：**The metabolism of sulforaphane (SFN): With different environmental temperature, PH and coenzyme factors, the glucoraphanin (GRP) could be hydrolyzed by the myrosinase into epithionitrile, isothiocyanates, thiocyanate, and nitrile. As one of the most reactive degradation products, isothiocyanate (ITC) can transform into SFN, which will be further metabolized through the mercapturic acid pathway to synthesize LSF-N-Acetyl-L-cysteine (LSF-NAC) and LSF-L-cysteine (LSF-cys).





**Supplemental** **Figure 2：**The mechanism and future development of SFN for eye diseases [By Figdraw.]: SFN can be applied in treating various ophthalmic diseases like AMD, DR, cataract, PCO, VKC, keratoconus, retinal degeneration, retinal ischemic injury, FECD and photoreceptor degeneration. The protective effects of SFN have been linked to mechanism including Nrf2 antioxidative pathway, NF-κB pathway, AMPK pathway, Txnip/mTOR pathway, and Nrf-2/HO-1 antioxidant pathway.


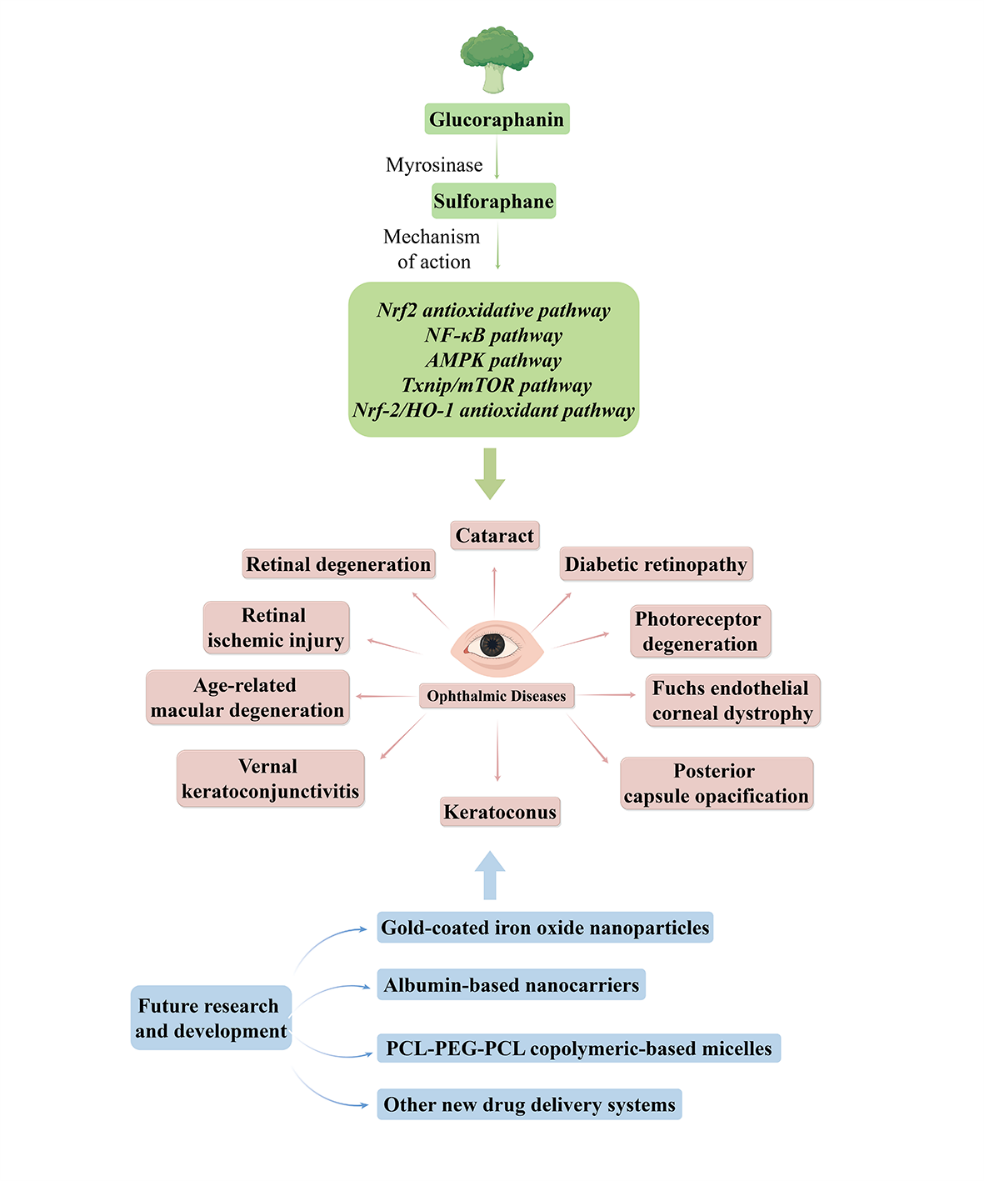


**Supplemental Table 1：**Physicochemical properties of SFN.

| Property Name | Property Value |
| --- | --- |
| Molecular Formula | C_6_H_11_NOS_2_ |
| Canonical SMILES | CS(=O)CCCCN=C=S |
| IUPAC Name | 1-isothiocyanato-4-methylsulfinylbutane |
| InChI | InChI=1S/C6H11NOS2/c1-10(8)5-3-2-4-7-6-9/h2-5H2,1H3 |
| Molecular Weight | 177.3g/mol |
| UNII Number | 41684WL1GL |
| CAS Number | 4478-93-7 |
| Physical Description | Solid |
| Melting point | 74.6°C |
| Boiling Point | 368.00 to 369.00 °C |

**Supplemental Table 2：**Biological activity and mechanism of action of SFN.

| Biological Activity | Effect | Mechanism of Action | Reference |
| --- | --- | --- | --- |
| Anti-diabetic | Reduce glucose production, prevent diabetes induced cardiomyopathy, renal injury and skeletal muscle dysfunction | AMPK mediated activation of Nrf2 antioxidative and renal lipid metabolic pathways | [15] |
| Anti-inflammatory | Reduce levels of inflammatory mediators and proinflammatory cytokines | Upregulate Nrf2 and HO-1 pathways, downregulate NF-κB, MAPK, STAT6, AP-1 pathways | [19-20] |
| Antimicrobial | Inhibit bacterial pathogen growth, reduce bacterial infections, kill bacteria, block gastric tumor formation | Destroy cell membrane integrity, inhibit enzymes involved in redox balance and bacteria metabolism | [24] |
| Anti-angiogenic | Inhibit proliferative ability, cell viability, migration, adhesion and tube formation | Induct apoptosis, activate FOXO transcription factors and inhibit STAT3/HIF-1α/VEGF signaling | [28,34] |
| Anticancer | Promote apoptosis, inhibit cell viability, proliferation, metastasis, malignancy, and epithelial-to-mesenchymal transition | Downregulate Wnt/β-catenin, RAF/MEK/ERK and NF-κB pathways | [35-37] |
| Anti-oxidant | Activate cellular antioxidant enzymes | Upregulate Nrf2-ARE signal pathway | [44] |

**Supplemental Table 3：**The effects and mechanism of action of SFN in ophthalmic diseases treatment.

| Ophthalmic Diseases | Effect | Mechanism of Action | Reference |
| --- | --- | --- | --- |
| AMD | Improve antioxidative ability of RPE 19 cells, reduce retinal light damage in RPE and photoreceptor cells, reverse pro-apoptotic changes | Increase the expression of antioxidative genes, Trx and phase II enzymes, downregulate inflammatory response genes, decrease oxidative stress | [50-52] |
| DR | Retard autophagy and neurodegeneration in retinal photoreceptor cells | Reduce oxidative stress, AGEs accumulation, inflammasome activation and inflammation | [58, 59, 63] |
| Cataract | Intervene the wound-healing in lens cells, protect aging lens epithelial cells. | Downregulate glutathione reductase activity, upregulate the activity of Nrf2/ARE/Prdx6 peroxiredoxin, activate cellular antioxidant enzymes | [68, 70] |
| VKC | Inhibit expressions of chemokine and adhesion molecule in human corneal fibroblasts | Reduce phosphorylation levels of MAPKs, IκBα and STAT6 | [20] |
| Keratoconus | Protects corneas against oxidative stress injury | Activate Nrf-2/HO-1 antioxidant pathway | [73] |
| Retinitis pigmentosa | Reduce photoreceptor apoptosis and retinal degeneration | Inhibit GRP78/BiP expression | [74] |
| Retinal ischemic injury | Alleviated ischemic induced retinal dysfunction and inner retinal layers attenuation | Activate Nrf2/HO-1 antioxidant pathway | [75, 76] |
| FECD | Reduce apoptosis of corneal endothelial cells | Upregulate Nrf2-ARE pathway | [77] |
| Retinal dystrophic syndromes | Retard photoreceptor degeneration | Increase levels of Nrf2, Trx and TrxR | [78] |

* SFN: sulforaphane; AMD: age-related macular degeneration; DR: diabetic retinopathy; RPE-19 cells: retinal pigment epithelium 19 cells; AGEs: advanced glycation end products; VKC: vernal keratoconjunctivitis; FECD: fuchs endothelial corneal dystrophy.

**Supplemental Table 4：**Recent clinical trials of SFN (ongoing and completed).

| NCT Number | Disease | Study Type | Phases | Study Design | Age | Enrollment | Interventions | Study Status | Reference |
| --- | --- | --- | --- | --- | --- | --- | --- | --- | --- |
| NCT02909959 | Autism Spectrum Disorder | Interventional | Phase 2 | Randomized | 13-30 | 48 | Avmacol^®^ 3-8 tablets orally daily, each tablet providing approximately 15 µmol SFN | May 2019 (COMPLETED) | (University of North Carolina, Translational, & Institute, 2017) |
| NCT02656420 | Environmental Carcinogenesis | Interventional | Phase 1 Phase 2 | Randomized | 21-65 | 170 | Maximum, half and one-fifth doses of broccoli sprout-derived beverage | March 2016 (COMPLETED) | (Health et al., 2016) |
| NCT02810964 | Schizophrenia | Interventional | Phase 2 | Randomized | 18-65 | 64 | Avmacol^®^ 6 tablets orally daily | November 2019 (COMPLETED) | (System, 2017) |
| NCT02561481 | Autism Spectrum Disorder | Interventional | Phase 1 Phase 2 | Randomized | 3-12 | 60 | Tablet will be administered once a day orally containing broccoli seed powder equivalent to 45-120 µmol SFN | January 2020 (COMPLETED)5 | (University of Massachusetts et al., 2015) |
| NCT03232138 | Lung Cancer | Interventional | Phase 2 | Randomized | 55–75 | 67 | SFN four tablets 2 times per day with breakfast and dinner each dose contains approximately 120 μmol of SFN | October 2023 (ONGOING) | (Jian-Min Yuan, Institute, & Pittsburgh, 2018) |
| NCT02677051 | Autism | Interventional | Phase 2 | Randomized | 13-30 | 48 | Oral pills containing broccoli seed powder equal to 50– 150 μmol SFN for 22 weeks | July 2024 (ONGOING) | (Rutgers & University, 2016) |
| NCT03665922 | Prostate Cancer | Interventional | NA | Randomized | 18-90 | 39 | Oral four BroccoMax^®^ tablets with breakfast and four tablets with dinner. The eight BroccoMax^®^ tablets will provide a daily internal dose of 64 mg of SFN | December 2024 (ONGOING) | (Pittsburgh & Institute, 2019) |
| NCT02614742 | Subarachnoid Hemorrhage, Spontaneous | Interventional | Phase 2 | Randomized | 18-80 | 90 | Sulforadex^®^ 300mg bid for up to 28 days | November 2019 (COMPLETED) | (Pharma, 2016) |
| NCT05797506 | Chronic Kidney Disease Stage 3-4 | Interventional | Phase 2 | Randomized | 18-80 | 100 | Oral 4 Tablets of SFN (Avmacol Extra Strength) per day in patients with chronic kidney disease, stages 3-4 | December 2025 (ONGOING) | (Rochester et al., 2023) |
| NCT05408559 | Diastolic Dysfunction | Interventional | Phase 1 Phase 2 | Randomized | 60-80 | 200 | Oral 2-4 caplets containing SFN-rich broccoli sprout extracts (Avmacol Extra Strength) for 24 weeks | July 2026 (ONGOING) | (Center, 2022) |
| NCT03932136 | Clinical High Risk Syndrome of Psychosis | Interventional | Phase 3 | Randomized | 15-45 | 300 | Oral 6 tablets containing SFN per day for 52 weeks | December 2026 (ONGOING) | (Medicine et al., 2019) |
